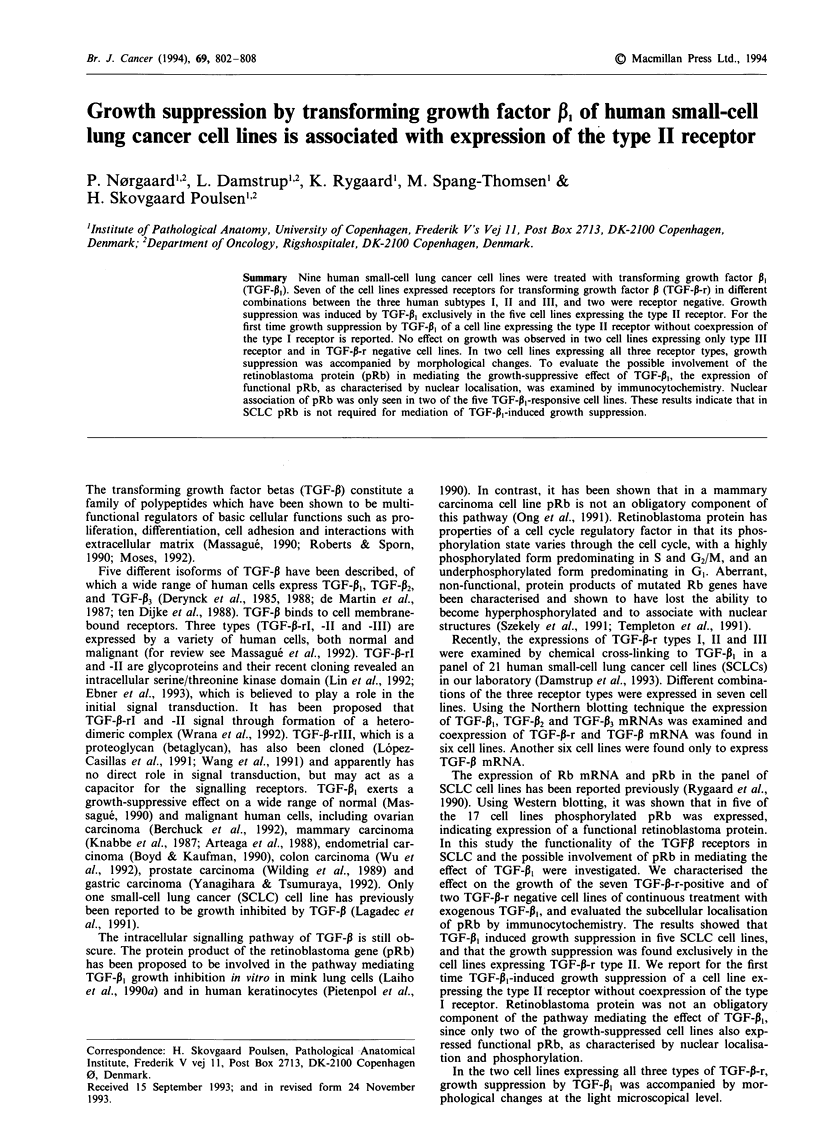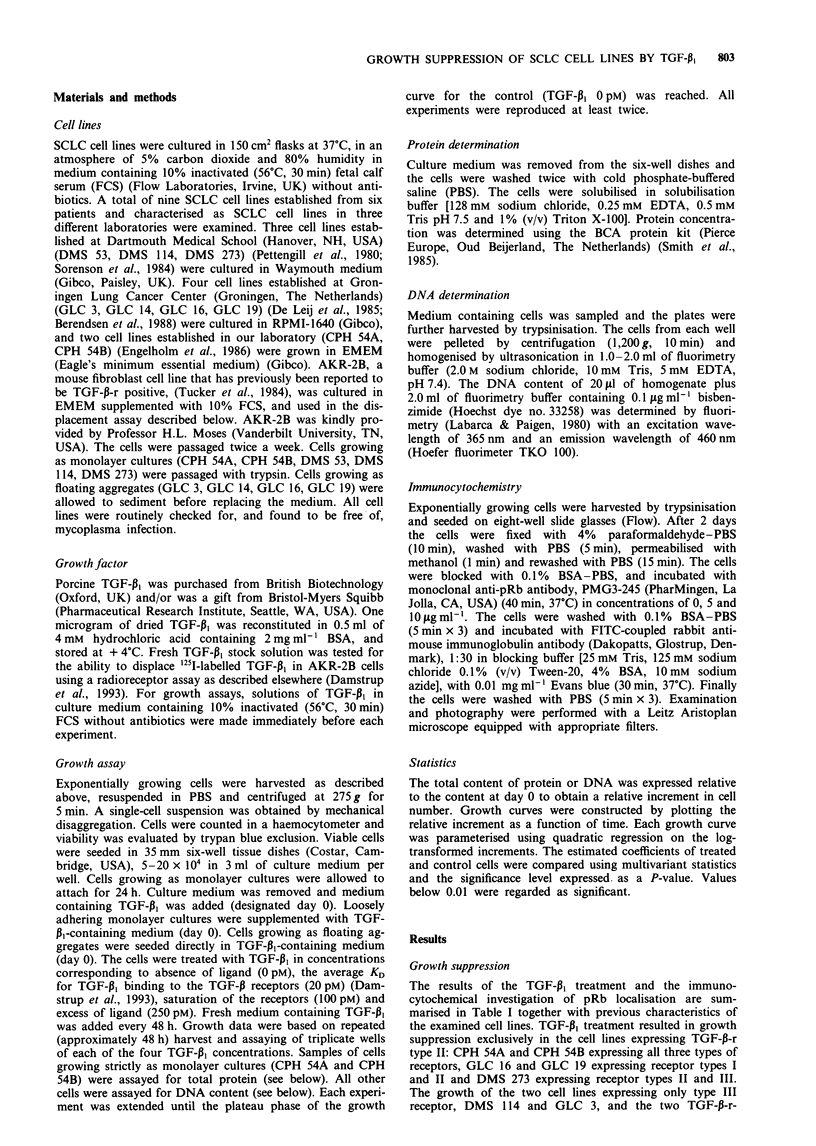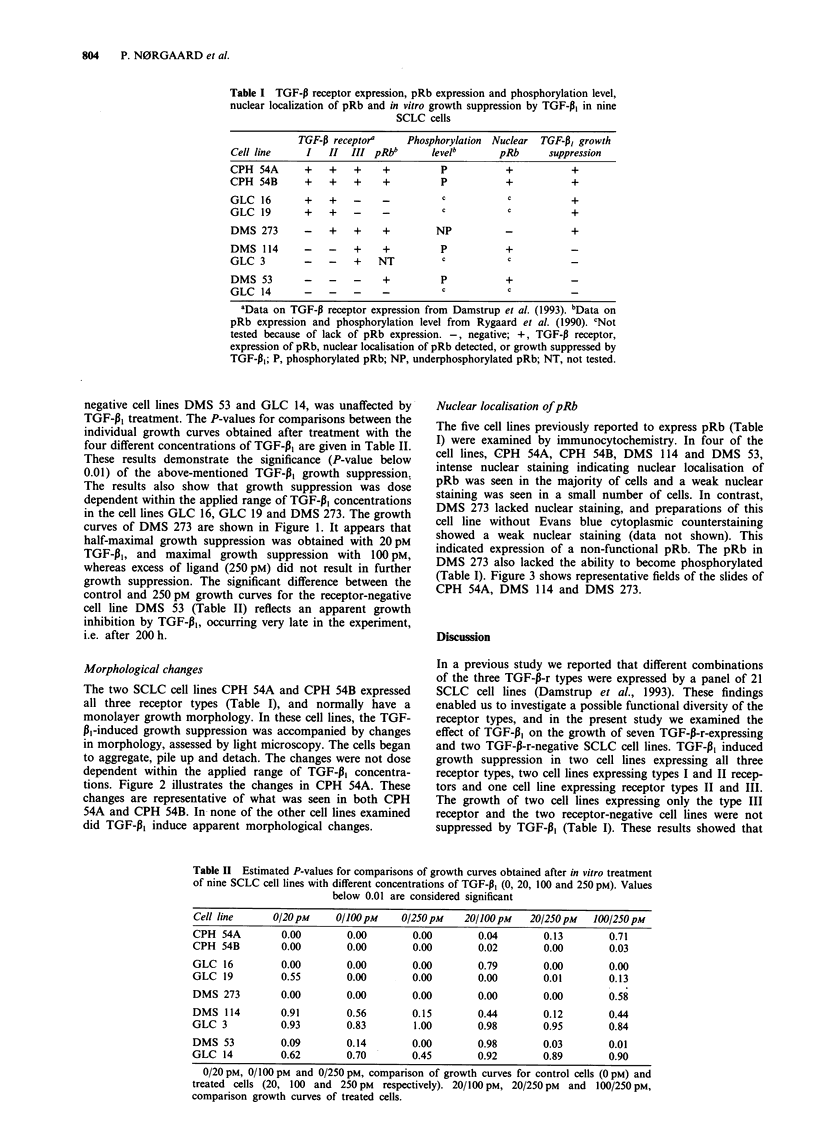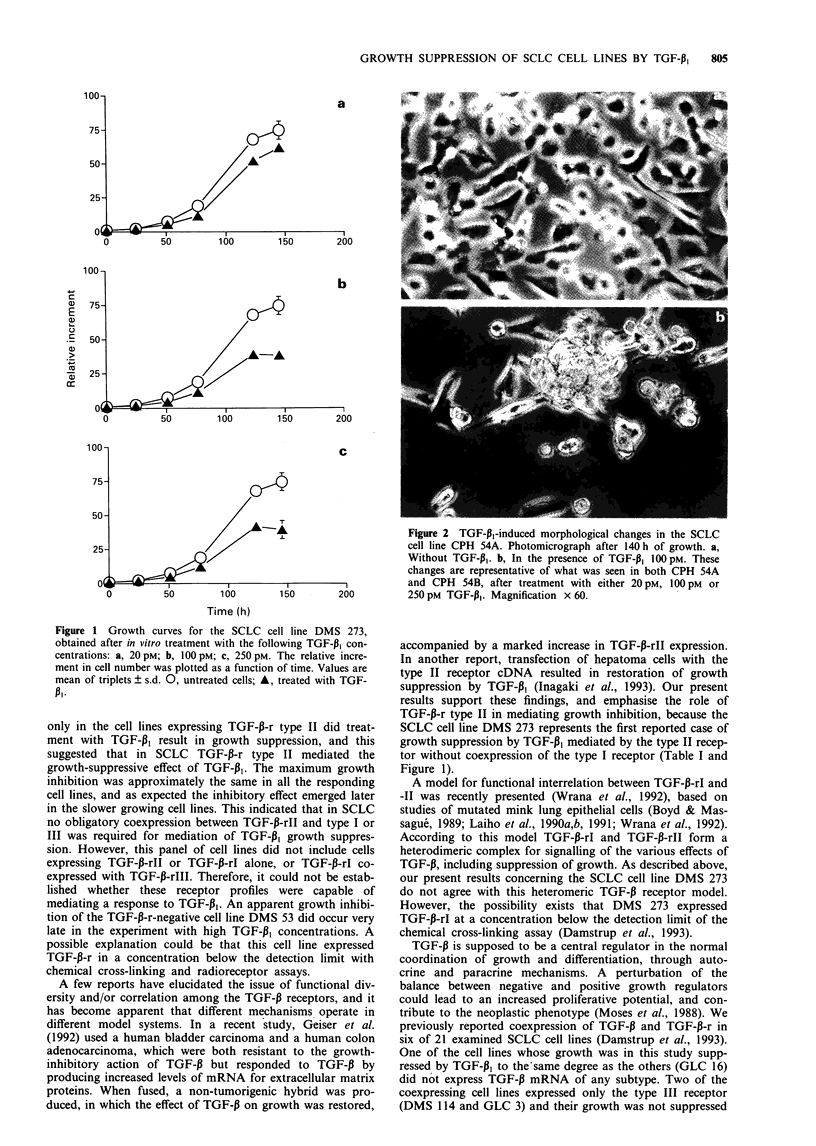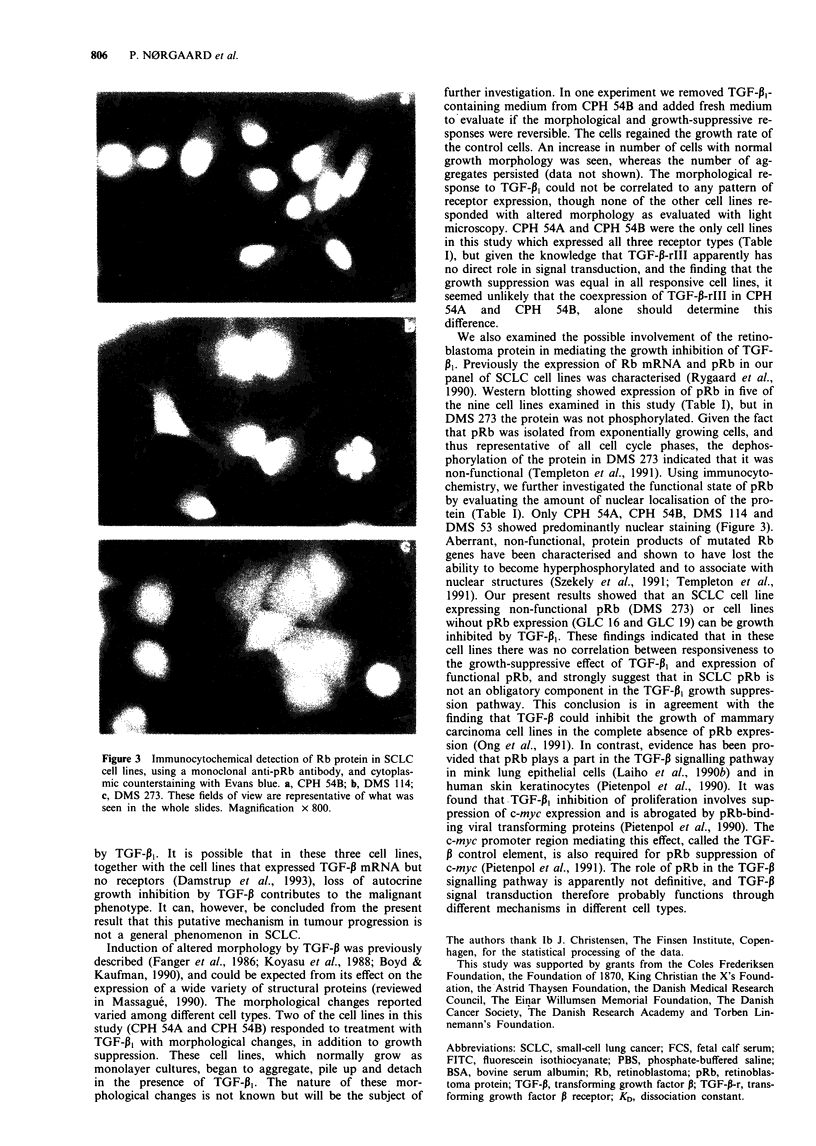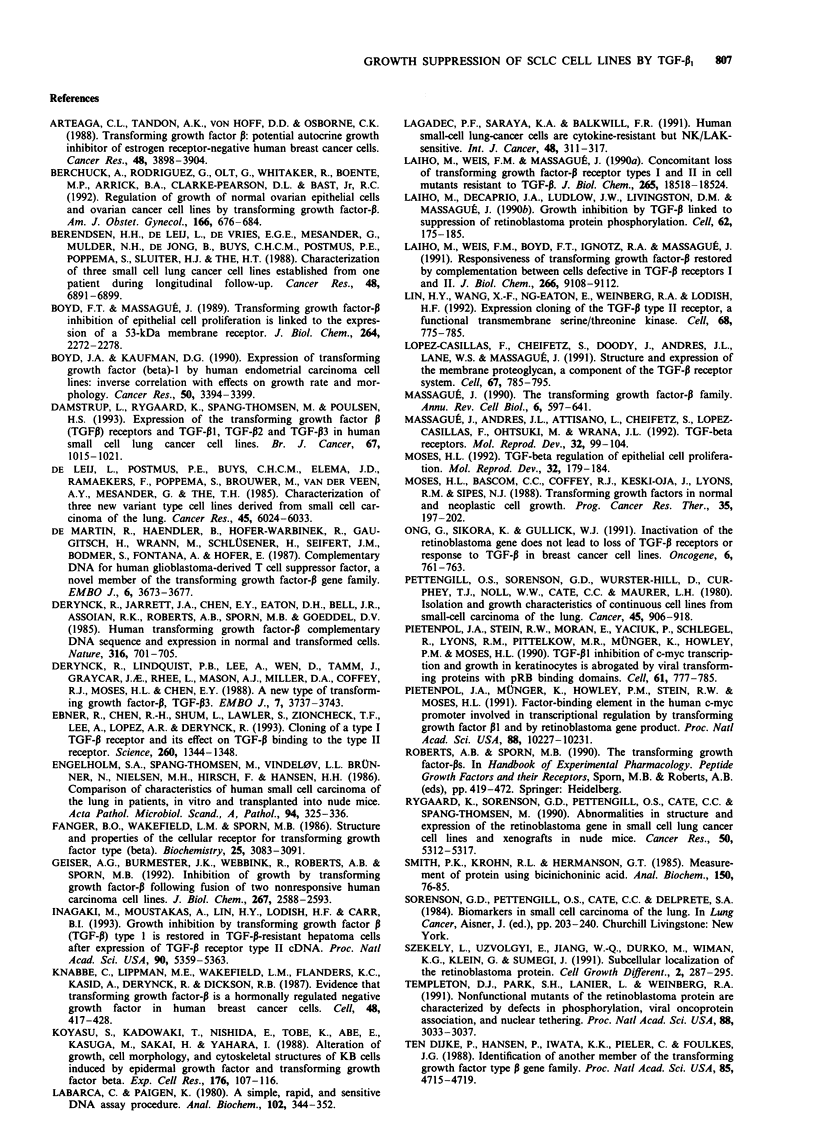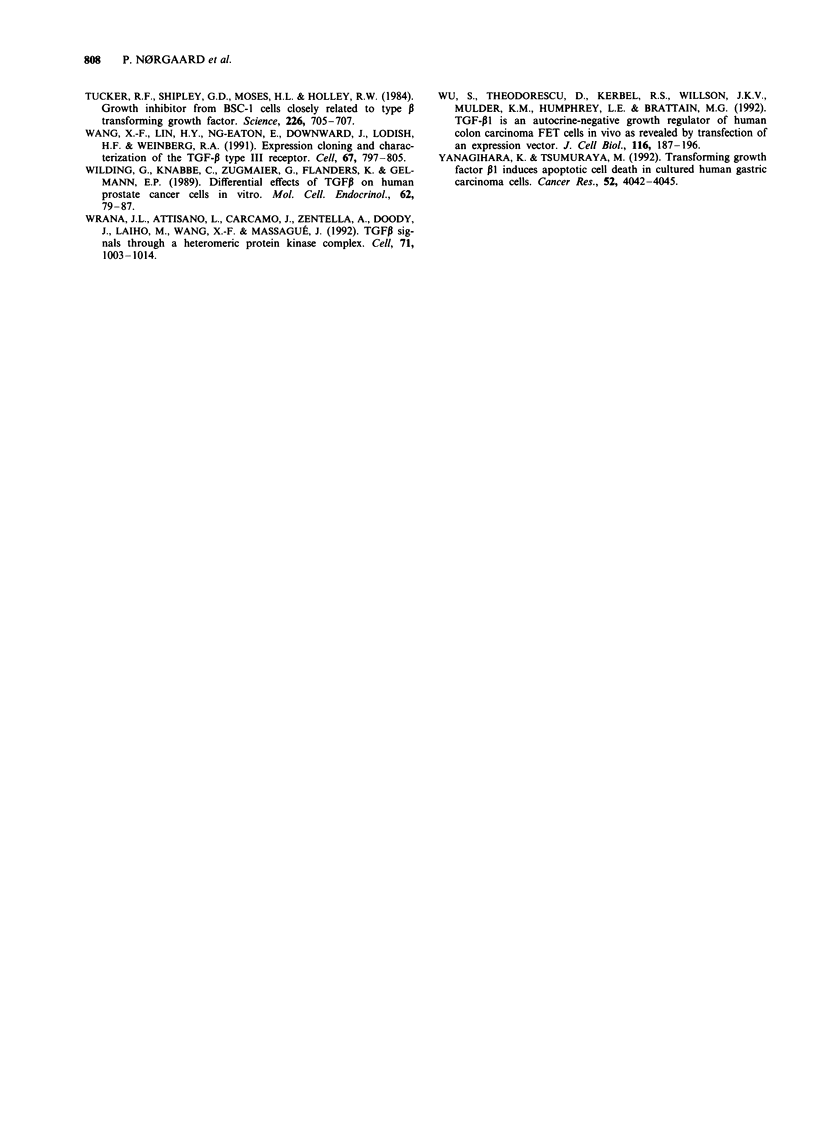# Growth suppression by transforming growth factor beta 1 of human small-cell lung cancer cell lines is associated with expression of the type II receptor.

**DOI:** 10.1038/bjc.1994.158

**Published:** 1994-05

**Authors:** P. Nørgaard, L. Damstrup, K. Rygaard, M. Spang-Thomsen, H. Skovgaard Poulsen

**Affiliations:** Institute of Pathological Anatomy, University of Copenhagen, Denmark.

## Abstract

**Images:**


*Br. J. Cancer* (1994), **69**, 802–808 © Macmillan Press Ltd., 1994

### Growth suppression by transforming growth factor $\beta_1$ of human small-cell lung cancer cell lines is associated with expression of the type II receptor

P. Nørgaard[1,2], L. Damstrup[1,2], K. Rygaard[1], M. Spang-Thomsen[1] & H. Skovgaard Poulsen[1,2]

[1]*Institute of Pathological Anatomy, University of Copenhagen, Frederik V's Vej 11, Post Box 2713, DK-2100 Copenhagen, Denmark;* [2]*Department of Oncology, Rigshospitalet, DK-2100 Copenhagen, Denmark.*

**Summary** Nine human small-cell lung cancer cell lines were treated with transforming growth factor $\beta_1$ (TGF-$\beta_1$). Seven of the cell lines expressed receptors for transforming growth factor $\beta$ (TGF-$\beta$-r) in different combinations between the three human subtypes I, II and III, and two were receptor negative. Growth suppression was induced by TGF-$\beta_1$ exclusively in the five cell lines expressing the type II receptor. For the first time growth suppression by TGF-$\beta_1$ of a cell line expressing the type II receptor without coexpression of the type I receptor is reported. No effect on growth was observed in two cell lines expressing only type III receptor and in TGF-$\beta$-r negative cell lines. In two cell lines expressing all three receptor types, growth suppression was accompanied by morphological changes. To evaluate the possible involvement of the retinoblastoma protein (pRb) in mediating the growth-suppressive effect of TGF-$\beta_1$, the expression of functional pRb, as characterised by nuclear localisation, was examined by immunocytochemistry. Nuclear association of pRb was only seen in two of the five TGF-$\beta_1$-responsive cell lines. These results indicate that in SCLC pRb is not required for mediation of TGF-$\beta_1$-induced growth suppression.

The transforming growth factor betas (TGF-$\beta$) constitute a family of polypeptides which have been shown to be multifunctional regulators of basic cellular functions such as proliferation, differentiation, cell adhesion and interactions with extracellular matrix (Massagué, 1990; Roberts & Sporn, 1990; Moses, 1992).

Five different isoforms of TGF-$\beta$ have been described, of which a wide range of human cells express TGF-$\beta_1$, TGF-$\beta_2$, and TGF-$\beta_3$ (Derynck *et al.*, 1985, 1988; de Martin *et al.*, 1987; ten Dijke *et al.*, 1988). TGF-$\beta$ binds to cell membrane-bound receptors. Three types (TGF-$\beta$-rI, -II and -III) are expressed by a variety of human cells, both normal and malignant (for review see Massagué *et al.*, 1992). TGF-$\beta$-rI and -II are glycoproteins and their recent cloning revealed an intracellular serine/threonine kinase domain (Lin *et al.*, 1992; Ebner *et al.*, 1993), which is believed to play a role in the initial signal transduction. It has been proposed that TGF-$\beta$-rI and -II signal through formation of a heterodimeric complex (Wrana *et al.*, 1992). TGF-$\beta$-rIII, which is a proteoglycan (betaglycan), has also been cloned (López-Casillas *et al.*, 1991; Wang *et al.*, 1991) and apparently has no direct role in signal transduction, but may act as a capacitor for the signalling receptors. TGF-$\beta_1$ exerts a growth-suppressive effect on a wide range of normal (Massagué, 1990) and malignant human cells, including ovarian carcinoma (Berchuck *et al.*, 1992), mammary carcinoma (Knabbe *et al.*, 1987; Arteaga *et al.*, 1988), endometrial carcinoma (Boyd & Kaufman, 1990), colon carcinoma (Wu *et al.*, 1992), prostate carcinoma (Wilding *et al.*, 1989) and gastric carcinoma (Yanagihara & Tsumuraya, 1992). Only one small-cell lung cancer (SCLC) cell line has previously been reported to be growth inhibited by TGF-$\beta$ (Lagadec *et al.*, 1991).

The intracellular signalling pathway of TGF-$\beta$ is still obscure. The protein product of the retinoblastoma gene (pRb) has been proposed to be involved in the pathway mediating TGF-$\beta_1$ growth inhibition *in vitro* in mink lung cells (Laiho *et al.*, 1990*a*) and in human keratinocytes (Pietenpol *et al.*, 1990). In contrast, it has been shown that in a mammary carcinoma cell line pRb is not an obligatory component of this pathway (Ong *et al.*, 1991). Retinoblastoma protein has properties of a cell cycle regulatory factor in that its phosphorylation state varies through the cell cycle, with a highly phosphorylated form predominating in S and $G_2$/M, and an underphosphorylated form predominating in $G_1$. Aberrant, non-functional, protein products of mutated Rb genes have been characterised and shown to have lost the ability to become hyperphosphorylated and to associate with nuclear structures (Szekely *et al.*, 1991; Templeton *et al.*, 1991).

Recently, the expressions of TGF-$\beta$-r types I, II and III were examined by chemical cross-linking to TGF-$\beta_1$ in a panel of 21 human small-cell lung cancer cell lines (SCLCs) in our laboratory (Damstrup *et al.*, 1993). Different combinations of the three receptor types were expressed in seven cell lines. Using the Northern blotting technique the expression of TGF-$\beta_1$, TGF-$\beta_2$ and TGF-$\beta_3$ mRNAs was examined and coexpression of TGF-$\beta$-r and TGF-$\beta$ mRNA was found in six cell lines. Another six cell lines were found only to express TGF-$\beta$ mRNA.

The expression of Rb mRNA and pRb in the panel of SCLC cell lines has been reported previously (Rygaard *et al.*, 1990). Using Western blotting, it was shown that in five of the 17 cell lines phosphorylated pRb was expressed, indicating expression of a functional retinoblastoma protein. In this study the functionality of the TGF$\beta$ receptors in SCLC and the possible involvement of pRb in mediating the effect of TGF-$\beta_1$ were investigated. We characterised the effect on the growth of the seven TGF-$\beta$-r-positive and of two TGF-$\beta$-r negative cell lines of continuous treatment with exogenous TGF-$\beta_1$, and evaluated the subcellular localisation of pRb by immunocytochemistry. The results showed that TGF-$\beta_1$ induced growth suppression in five SCLC cell lines, and that the growth suppression was found exclusively in the cell lines expressing TGF-$\beta$-r type II. We report for the first time TGF-$\beta_1$-induced growth suppression of a cell line expressing the type II receptor without coexpression of the type I receptor. Retinoblastoma protein was not an obligatory component of the pathway mediating the effect of TGF-$\beta_1$, since only two of the growth-suppressed cell lines also expressed functional pRb, as characterised by nuclear localisation and phosphorylation.

In the two cell lines expressing all three types of TGF-$\beta$-r, growth suppression by TGF-$\beta_1$ was accompanied by morphological changes at the light microscopical level.

Correspondence: H. Skovgaard Poulsen, Pathological Anatomical Institute, Frederik V vej 11, Post Box 2713, DK-2100 Copenhagen Ø, Denmark.
Received 15 September 1993; and in revised form 24 November 1993.

### Materials and methods

### Cell lines

SCLC cell lines were cultured in 150 $cm^2$ flasks at 37°C, in an atmosphere of 5% carbon dioxide and 80% humidity in medium containing 10% inactivated (56°C, 30 min) fetal calf serum (FCS) (Flow Laboratories, Irvine, UK) without antibiotics. A total of nine SCLC cell lines established from six patients and characterised as SCLC cell lines in three different laboratories were examined. Three cell lines established at Dartmouth Medical School (Hanover, NH, USA) (DMS 53, DMS 114, DMS 273) (Pettengill *et al.*, 1980; Sorenson *et al.*, 1984) were cultured in Waymouth medium (Gibco, Paisley, UK). Four cell lines established at Groningen Lung Cancer Center (Groningen, The Netherlands) (GLC 3, GLC 14, GLC 16, GLC 19) (De Leij *et al.*, 1985; Berendsen *et al.*, 1988) were cultured in RPMI-1640 (Gibco), and two cell lines established in our laboratory (CPH 54A, CPH 54B) (Engelholm *et al.*, 1986) were grown in EMEM (Eagle's minimum essential medium) (Gibco). AKR-2B, a mouse fibroblast cell line that has previously been reported to be TGF-$\beta$-r positive, (Tucker *et al.*, 1984), was cultured in EMEM supplemented with 10% FCS, and used in the displacement assay described below. AKR-2B was kindly provided by Professor H.L. Moses (Vanderbilt University, TN, USA). The cells were passaged twice a week. Cells growing as monolayer cultures (CPH 54A, CPH 54B, DMS 53, DMS 114, DMS 273) were passaged with trypsin. Cells growing as floating aggregates (GLC 3, GLC 14, GLC 16, GLC 19) were allowed to sediment before replacing the medium. All cell lines were routinely checked for, and found to be free of, mycoplasma infection.

### Growth factor

Porcine TGF-$\beta_1$ was purchased from British Biotechnology (Oxford, UK) and/or was a gift from Bristol-Myers Squibb (Pharmaceutical Research Institute, Seattle, WA, USA). One microgram of dried TGF-$\beta_1$ was reconstituted in 0.5 ml of 4 mM hydrochloric acid containing 2 mg $ml^{-1}$ BSA, and stored at + 4°C. Fresh TGF-$\beta_1$ stock solution was tested for the ability to displace $^{125}$I-labelled TGF-$\beta_1$ in AKR-2B cells using a radioreceptor assay as described elsewhere (Damstrup *et al.*, 1993). For growth assays, solutions of TGF-$\beta_1$ in culture medium containing 10% inactivated (56°C, 30 min) FCS without antibiotics were made immediately before each experiment.

### Growth assay

Exponentially growing cells were harvested as described above, resuspended in PBS and centrifuged at 275 *g* for 5 min. A single-cell suspension was obtained by mechanical disaggregation. Cells were counted in a haemocytometer and viability was evaluated by trypan blue exclusion. Viable cells were seeded in 35 mm six-well tissue dishes (Costar, Cambridge, USA), $5-20 \times 10^4$ in 3 ml of culture medium per well. Cells growing as monolayer cultures were allowed to attach for 24 h. Culture medium was removed and medium containing TGF-$\beta_1$ was added (designated day 0). Loosely adhering monolayer cultures were supplemented with TGF-$\beta_1$-containing medium (day 0). Cells growing as floating aggregates were seeded directly in TGF-$\beta_1$-containing medium (day 0). The cells were treated with TGF-$\beta_1$ in concentrations corresponding to absence of ligand (0 pM), the average $K_D$ for TGF-$\beta_1$ binding to the TGF-$\beta$ receptors (20 pM) (Damstrup *et al.*, 1993), saturation of the receptors (100 pM) and excess of ligand (250 pM). Fresh medium containing TGF-$\beta_1$ was added every 48 h. Growth data were based on repeated (approximately 48 h) harvest and assaying of triplicate wells of each of the four TGF-$\beta_1$ concentrations. Samples of cells growing strictly as monolayer cultures (CPH 54A and CPH 54B) were assayed for total protein (see below). All other cells were assayed for DNA content (see below). Each experiment was extended until the plateau phase of the growth curve for the control (TGF-$\beta_1$ 0 pM) was reached. All experiments were reproduced at least twice.

### Protein determination

Culture medium was removed from the six-well dishes and the cells were washed twice with cold phosphate-buffered saline (PBS). The cells were solubilised in solubilisation buffer [128 mM sodium chloride, 0.25 mM EDTA, 0.5 mM Tris pH 7.5 and 1% (v/v) Triton X-100]. Protein concentration was determined using the BCA protein kit (Pierce Europe, Oud Beijerland, The Netherlands) (Smith *et al.*, 1985).

### DNA determination

Medium containing cells was sampled and the plates were further harvested by trypsinisation. The cells from each well were pelleted by centrifugation (1,200 *g*, 10 min) and homogenised by ultrasonication in 1.0–2.0 ml of fluorimetry buffer (2.0 M sodium chloride, 10 mM Tris, 5 mM EDTA, pH 7.4). The DNA content of 20 $\mu$l of homogenate plus 2.0 ml of fluorimetry buffer containing 0.1 $\mu$g $ml^{-1}$ bisbenzimide (Hoechst dye no. 33258) was determined by fluorimetry (Labarca & Paigen, 1980) with an excitation wavelength of 365 nm and an emission wavelength of 460 nm (Hoefer fluorimeter TKO 100).

### Immunocytochemistry

Exponentially growing cells were harvested by trypsinisation and seeded on eight-well slide glasses (Flow). After 2 days the cells were fixed with 4% paraformaldehyde–PBS (10 min), washed with PBS (5 min), permeabilised with methanol (1 min) and rewashed with PBS (15 min). The cells were blocked with 0.1% BSA–PBS, and incubated with monoclonal anti-pRb antibody, PMG3-245 (PharMingen, La Jolla, CA, USA) (40 min, 37°C) in concentrations of 0, 5 and 10 $\mu$g $ml^{-1}$. The cells were washed with 0.1% BSA–PBS (5 min × 3) and incubated with FITC-coupled rabbit anti-mouse immunoglobulin antibody (Dakopatts, Glostrup, Denmark), 1:30 in blocking buffer [25 mM Tris, 125 mM sodium chloride 0.1% (v/v) Tween-20, 4% BSA, 10 mM sodium azide], with 0.01 mg $ml^{-1}$ Evans blue (30 min, 37°C). Finally the cells were washed with PBS (5 min × 3). Examination and photography were performed with a Leitz Aristoplan microscope equipped with appropriate filters.

### Statistics

The total content of protein or DNA was expressed relative to the content at day 0 to obtain a relative increment in cell number. Growth curves were constructed by plotting the relative increment as a function of time. Each growth curve was parameterised using quadratic regression on the log-transformed increments. The estimated coefficients of treated and control cells were compared using multivariant statistics and the significance level expressed as a *P*-value. Values below 0.01 were regarded as significant.

### Results

### Growth suppression

The results of the TGF-$\beta_1$ treatment and the immunocytochemical investigation of pRb localisation are summarised in Table I together with previous characteristics of the examined cell lines. TGF-$\beta_1$ treatment resulted in growth suppression exclusively in the cell lines expressing TGF-$\beta$-r type II: CPH 54A and CPH 54B expressing all three types of receptors, GLC 16 and GLC 19 expressing receptor types I and II and DMS 273 expressing receptor types II and III. The growth of the two cell lines expressing only type III receptor, DMS 114 and GLC 3, and the two TGF-$\beta$-r-

**Table I** TGF-β receptor expression, pRb expression and phosphorylation level, nuclear localization of pRb and *in vitro* growth suppression by TGF-$\beta_1$ in nine SCLC cells

| Cell line | TGF-β receptor[a] I | II | III | pRb[b] | Phosphorylation level[b] | Nuclear pRb | TGF-$\beta_1$ growth suppression |
|---|---|---|---|---|---|---|---|
| CPH 54A | + | + | + | + | P | + | + |
| CPH 54B | + | + | + | + | P | + | + |
| GLC 16 | + | + | − | − | c | c | + |
| GLC 19 | + | + | − | − | c | c | + |
| DMS 273 | − | + | + | + | NP | − | + |
| DMS 114 | − | − | + | + | P | + | − |
| GLC 3 | − | − | + | NT | c | c | − |
| DMS 53 | − | − | − | + | P | + | − |
| GLC 14 | − | − | − | − | c | c | − |

[a]Data on TGF-β receptor expression from Damstrup *et al.* (1993). [b]Data on pRb expression and phosphorylation level from Rygaard *et al.* (1990). [c]Not tested because of lack of pRb expression. −, negative; +, TGF-β receptor, expression of pRb, nuclear localisation of pRb detected, or growth suppressed by TGF-$\beta_1$; P, phosphorylated pRb; NP, underphosphorylated pRb; NT, not tested.

negative cell lines DMS 53 and GLC 14, was unaffected by TGF-$\beta_1$ treatment. The *P*-values for comparisons between the individual growth curves obtained after treatment with the four different concentrations of TGF-$\beta_1$ are given in Table II. These results demonstrate the significance (*P*-value below 0.01) of the above-mentioned TGF-$\beta_1$ growth suppression. The results also show that growth suppression was dose dependent within the applied range of TGF-$\beta_1$ concentrations in the cell lines GLC 16, GLC 19 and DMS 273. The growth curves of DMS 273 are shown in Figure 1. It appears that half-maximal growth suppression was obtained with 20 pM TGF-$\beta_1$, and maximal growth suppression with 100 pM, whereas excess of ligand (250 pM) did not result in further growth suppression. The significant difference between the control and 250 pM growth curves for the receptor-negative cell line DMS 53 (Table II) reflects an apparent growth inhibition by TGF-$\beta_1$, occurring very late in the experiment, i.e. after 200 h.

### *Morphological changes*

The two SCLC cell lines CPH 54A and CPH 54B expressed all three receptor types (Table I), and normally have a monolayer growth morphology. In these cell lines, the TGF-$\beta_1$-induced growth suppression was accompanied by changes in morphology, assessed by light microscopy. The cells began to aggregate, pile up and detach. The changes were not dose dependent within the applied range of TGF-$\beta_1$ concentrations. Figure 2 illustrates the changes in CPH 54A. These changes are representative of what was seen in both CPH 54A and CPH 54B. In none of the other cell lines examined did TGF-$\beta_1$ induce apparent morphological changes.

### *Nuclear localisation of pRb*

The five cell lines previously reported to express pRb (Table I) were examined by immunocytochemistry. In four of the cell lines, CPH 54A, CPH 54B, DMS 114 and DMS 53, intense nuclear staining indicating nuclear localisation of pRb was seen in the majority of cells and a weak nuclear staining was seen in a small number of cells. In contrast, DMS 273 lacked nuclear staining, and preparations of this cell line without Evans blue cytoplasmic counterstaining showed a weak nuclear staining (data not shown). This indicated expression of a non-functional pRb. The pRb in DMS 273 also lacked the ability to become phosphorylated (Table I). Figure 3 shows representative fields of the slides of CPH 54A, DMS 114 and DMS 273.

### Discussion

In a previous study we reported that different combinations of the three TGF-β-r types were expressed by a panel of 21 SCLC cell lines (Damstrup *et al.*, 1993). These findings enabled us to investigate a possible functional diversity of the receptor types, and in the present study we examined the effect of TGF-$\beta_1$ on the growth of seven TGF-β-r-expressing and two TGF-β-r-negative SCLC cell lines. TGF-$\beta_1$ induced growth suppression in two cell lines expressing all three receptor types, two cell lines expressing types I and II receptors and one cell line expressing receptor types II and III. The growth of two cell lines expressing only the type III receptor and the two receptor-negative cell lines were not suppressed by TGF-$\beta_1$ (Table I). These results showed that

**Table II** Estimated *P*-values for comparisons of growth curves obtained after *in vitro* treatment of nine SCLC cell lines with different concentrations of TGF-$\beta_1$ (0, 20, 100 and 250 pM). Values below 0.01 are considered significant

| Cell line | 0/20 pM | 0/100 pM | 0/250 pM | 20/100 pM | 20/250 pM | 100/250 pM |
|---|---|---|---|---|---|---|
| CPH 54A | 0.00 | 0.00 | 0.00 | 0.04 | 0.13 | 0.71 |
| CPH 54B | 0.00 | 0.00 | 0.00 | 0.02 | 0.00 | 0.03 |
| GLC 16 | 0.00 | 0.00 | 0.00 | 0.79 | 0.00 | 0.00 |
| GLC 19 | 0.55 | 0.00 | 0.00 | 0.00 | 0.01 | 0.13 |
| DMS 273 | 0.00 | 0.00 | 0.00 | 0.00 | 0.00 | 0.58 |
| DMS 114 | 0.91 | 0.56 | 0.15 | 0.44 | 0.12 | 0.44 |
| GLC 3 | 0.93 | 0.83 | 1.00 | 0.98 | 0.95 | 0.84 |
| DMS 53 | 0.09 | 0.14 | 0.00 | 0.98 | 0.03 | 0.01 |
| GLC 14 | 0.62 | 0.70 | 0.45 | 0.92 | 0.89 | 0.90 |

0/20 pM, 0/100 pM and 0/250 pM, comparison of growth curves for control cells (0 pM) and treated cells (20, 100 and 250 pM respectively). 20/100 pM, 20/250 pM and 100/250 pM, comparison growth curves of treated cells.

**Figure 1** Growth curves for the SCLC cell line DMS 273, obtained after *in vitro* treatment with the following TGF-$\beta_1$ concentrations: **a**, 20 pM; **b**, 100 pM; **c**, 250 pM. The relative increment in cell number was plotted as a function of time. Values are mean of triplets ± s.d. O, untreated cells; ▲, treated with TGF-$\beta_1$.

**Figure 2** TGF-$\beta_1$-induced morphological changes in the SCLC cell line CPH 54A. Photomicrograph after 140 h of growth. **a**, Without TGF-$\beta_1$. **b**, In the presence of TGF-$\beta_1$ 100 pM. These changes are representative of what was seen in both CPH 54A and CPH 54B, after treatment with either 20 pM, 100 pM or 250 pM TGF-$\beta_1$. Magnification × 60.

only in the cell lines expressing TGF-$\beta$-r type II did treatment with TGF-$\beta_1$ result in growth suppression, and this suggested that in SCLC TGF-$\beta$-r type II mediated the growth-suppressive effect of TGF-$\beta_1$. The maximum growth inhibition was approximately the same in all the responding cell lines, and as expected the inhibitory effect emerged later in the slower growing cell lines. This indicated that in SCLC no obligatory coexpression between TGF-$\beta$-rII and type I or III was required for mediation of TGF-$\beta_1$ growth suppression. However, this panel of cell lines did not include cells expressing TGF-$\beta$-rII or TGF-$\beta$-rI alone, or TGF-$\beta$-rI coexpressed with TGF-$\beta$-rIII. Therefore, it could not be established whether these receptor profiles were capable of mediating a response to TGF-$\beta_1$. An apparent growth inhibition of the TGF-$\beta$-r-negative cell line DMS 53 did occur very late in the experiment with high TGF-$\beta_1$ concentrations. A possible explanation could be that this cell line expressed TGF-$\beta$-r in a concentration below the detection limit with chemical cross-linking and radioreceptor assays.

A few reports have elucidated the issue of functional diversity and/or correlation among the TGF-$\beta$ receptors, and it has become apparent that different mechanisms operate in different model systems. In a recent study, Geiser *et al.* (1992) used a human bladder carcinoma and a human colon adenocarcinoma, which were both resistant to the growth-inhibitory action of TGF-$\beta$ but responded to TGF-$\beta$ by producing increased levels of mRNA for extracellular matrix proteins. When fused, a non-tumorigenic hybrid was produced, in which the effect of TGF-$\beta$ on growth was restored, accompanied by a marked increase in TGF-$\beta$-rII expression. In another report, transfection of hepatoma cells with the type II receptor cDNA resulted in restoration of growth suppression by TGF-$\beta_1$ (Inagaki *et al.*, 1993). Our present results support these findings, and emphasise the role of TGF-$\beta$-r type II in mediating growth inhibition, because the SCLC cell line DMS 273 represents the first reported case of growth suppression by TGF-$\beta_1$ mediated by the type II receptor without coexpression of the type I receptor (Table I and Figure 1).

A model for functional interrelation between TGF-$\beta$-rI and -II was recently presented (Wrana *et al.*, 1992), based on studies of mutated mink lung epithelial cells (Boyd & Massagué, 1989; Laiho *et al.*, 1990*a*,*b*, 1991; Wrana *et al.*, 1992). According to this model TGF-$\beta$-rI and TGF-$\beta$-rII form a heterodimeric complex for signalling of the various effects of TGF-$\beta$, including suppression of growth. As described above, our present results concerning the SCLC cell line DMS 273 do not agree with this heteromeric TGF-$\beta$ receptor model. However, the possibility exists that DMS 273 expressed TGF-$\beta$-rI at a concentration below the detection limit of the chemical cross-linking assay (Damstrup *et al.*, 1993).

TGF-$\beta$ is supposed to be a central regulator in the normal coordination of growth and differentiation, through autocrine and paracrine mechanisms. A perturbation of the balance between negative and positive growth regulators could lead to an increased proliferative potential, and contribute to the neoplastic phenotype (Moses *et al.*, 1988). We previously reported coexpression of TGF-$\beta$ and TGF-$\beta$-r in six of 21 examined SCLC cell lines (Damstrup *et al.*, 1993). One of the cell lines whose growth was in this study suppressed by TGF-$\beta_1$ to the same degree as the others (GLC 16) did not express TGF-$\beta$ mRNA of any subtype. Two of the coexpressing cell lines expressed only the type III receptor (DMS 114 and GLC 3) and their growth was not suppressed

**Figure 3** Immunocytochemical detection of Rb protein in SCLC cell lines, using a monoclonal anti-pRb antibody, and cytoplasmic counterstaining with Evans blue. **a**, CPH 54B; **b**, DMS 114; **c**, DMS 273. These fields of view are representative of what was seen in the whole slides. Magnification × 800.

by TGF-$\beta_1$. It is possible that in these three cell lines, together with the cell lines that expressed TGF-$\beta$ mRNA but no receptors (Damstrup *et al.*, 1993), loss of autocrine growth inhibition by TGF-$\beta$ contributes to the malignant phenotype. It can, however, be concluded from the present result that this putative mechanism in tumour progression is not a general phenomenon in SCLC.

Induction of altered morphology by TGF-$\beta$ was previously described (Fanger *et al.*, 1986; Koyasu *et al.*, 1988; Boyd & Kaufman, 1990), and could be expected from its effect on the expression of a wide variety of structural proteins (reviewed in Massagué, 1990). The morphological changes reported varied among different cell types. Two of the cell lines in this study (CPH 54A and CPH 54B) responded to treatment with TGF-$\beta_1$ with morphological changes, in addition to growth suppression. These cell lines, which normally grow as monolayer cultures, began to aggregate, pile up and detach in the presence of TGF-$\beta_1$. The nature of these morphological changes is not known but will be the subject of further investigation. In one experiment we removed TGF-$\beta_1$-containing medium from CPH 54B and added fresh medium to evaluate if the morphological and growth-suppressive responses were reversible. The cells regained the growth rate of the control cells. An increase in number of cells with normal growth morphology was seen, whereas the number of aggregates persisted (data not shown). The morphological response to TGF-$\beta_1$ could not be correlated to any pattern of receptor expression, though none of the other cell lines responded with altered morphology as evaluated with light microscopy. CPH 54A and CPH 54B were the only cell lines in this study which expressed all three receptor types (Table I), but given the knowledge that TGF-$\beta$-rIII apparently has no direct role in signal transduction, and the finding that the growth suppression was equal in all responsive cell lines, it seemed unlikely that the coexpression of TGF-$\beta$-rIII in CPH 54A and CPH 54B, alone should determine this difference.

We also examined the possible involvement of the retinoblastoma protein in mediating the growth inhibition of TGF-$\beta_1$. Previously the expression of Rb mRNA and pRb in our panel of SCLC cell lines was characterised (Rygaard *et al.*, 1990). Western blotting showed expression of pRb in five of the nine cell lines examined in this study (Table I), but in DMS 273 the protein was not phosphorylated. Given the fact that pRb was isolated from exponentially growing cells, and thus representative of all cell cycle phases, the dephosphorylation of the protein in DMS 273 indicated that it was non-functional (Templeton *et al.*, 1991). Using immunocytochemistry, we further investigated the functional state of pRb by evaluating the amount of nuclear localisation of the protein (Table I). Only CPH 54A, CPH 54B, DMS 114 and DMS 53 showed predominantly nuclear staining (Figure 3). Aberrant, non-functional, protein products of mutated Rb genes have been characterised and shown to have lost the ability to become hyperphosphorylated and to associate with nuclear structures (Szekely *et al.*, 1991; Templeton *et al.*, 1991). Our present results showed that an SCLC cell line expressing non-functional pRb (DMS 273) or cell lines wihout pRb expression (GLC 16 and GLC 19) can be growth inhibited by TGF-$\beta_1$. These findings indicated that in these cell lines there was no correlation between responsiveness to the growth-suppressive effect of TGF-$\beta_1$ and expression of functional pRb, and strongly suggest that in SCLC pRb is not an obligatory component in the TGF-$\beta_1$ growth suppression pathway. This conclusion is in agreement with the finding that TGF-$\beta$ could inhibit the growth of mammary carcinoma cell lines in the complete absence of pRb expression (Ong *et al.*, 1991). In contrast, evidence has been provided that pRb plays a part in the TGF-$\beta$ signalling pathway in mink lung epithelial cells (Laiho *et al.*, 1990*b*) and in human skin keratinocytes (Pietenpol *et al.*, 1990). It was found that TGF-$\beta_1$ inhibition of proliferation involves suppression of c-*myc* expression and is abrogated by pRb-binding viral transforming proteins (Pietenpol *et al.*, 1990). The c-*myc* promoter region mediating this effect, called the TGF-$\beta$ control element, is also required for pRb suppression of c-*myc* (Pietenpol *et al.*, 1991). The role of pRb in the TGF-$\beta$ signalling pathway is apparently not definitive, and TGF-$\beta$ signal transduction therefore probably functions through different mechanisms in different cell types.

The authors thank Ib J. Christensen, The Finsen Institute, Copenhagen, for the statistical processing of the data.

This study was supported by grants from the Coles Frederiksen Foundation, the Foundation of 1870, King Christian the X's Foundation, the Astrid Thaysen Foundation, the Danish Medical Research Council, The Einar Willumsen Memorial Foundation, The Danish Cancer Society, The Danish Research Academy and Torben Linnemann's Foundation.

Abbreviations: SCLC, small-cell lung cancer; FCS, fetal calf serum; FITC, fluorescein isothiocyanate; PBS, phosphate-buffered saline; BSA, bovine serum albumin; Rb, retinoblastoma; pRb, retinoblastoma protein; TGF-$\beta$, transforming growth factor $\beta$; TGF-$\beta$-r, transforming growth factor $\beta$ receptor; $K_D$, dissociation constant.

### References

ARTEAGA, C.L., TANDON, A.K., VON HOFF, D.D. & OSBORNE, C.K. (1988). Transforming growth factor β: potential autocrine growth inhibitor of estrogen receptor-negative human breast cancer cells. *Cancer Res.*, **48**, 3898–3904.

BERCHUCK, A., RODRIGUEZ, G., OLT, G., WHITAKER, R., BOENTE, M.P., ARRICK, B.A., CLARKE-PEARSON, D.L. & BAST, Jr, R.C. (1992). Regulation of growth of normal ovarian epithelial cells and ovarian cancer cell lines by transforming growth factor-β. *Am. J. Obstet. Gynecol.*, **166**, 676–684.

BERENDSEN, H.H., DE LEIJ, L., DE VRIES, E.G.E., MESANDER, G., MULDER, N.H., DE JONG, B., BUYS, C.H.C.M., POSTMUS, P.E., POPPEMA, S., SLUITER, H.J. & THE, H.T. (1988). Characterization of three small cell lung cancer cell lines established from one patient during longitudinal follow-up. *Cancer Res.*, **48**, 6891–6899.

BOYD, F.T. & MASSAGUÉ, J. (1989). Transforming growth factor-β inhibition of epithelial cell proliferation is linked to the expression of a 53-kDa membrane receptor. *J. Biol. Chem.*, **264**, 2272–2278.

BOYD, J.A. & KAUFMAN, D.G. (1990). Expression of transforming growth factor (beta)-1 by human endometrial carcinoma cell lines: inverse correlation with effects on growth rate and morphology. *Cancer Res.*, **50**, 3394–3399.

DAMSTRUP, L., RYGAARD, K., SPANG-THOMSEN, M. & POULSEN, H.S. (1993). Expression of the transforming growth factor β (TGFβ) receptors and TGF-β1, TGF-β2 and TGF-β3 in human small cell lung cancer cell lines. *Br. J. Cancer*, **67**, 1015–1021.

DE LEIJ, L., POSTMUS, P.E., BUYS, C.H.C.M., ELEMA, J.D., RAMAEKERS, F., POPPEMA, S., BROUWER, M., VAN DER VEEN, A.Y., MESANDER, G. & THE, T.H. (1985). Characterization of three new variant type cell lines derived from small cell carcinoma of the lung. *Cancer Res.*, **45**, 6024–6033.

DE MARTIN, R., HAENDLER, B., HOFER-WARBINEK, R., GAUGITSCH, H., WRANN, M., SCHLÜSENER, H., SEIFERT, J.M., BODMER, S., FONTANA, A. & HOFER, E. (1987). Complementary DNA for human glioblastoma-derived T cell suppressor factor, a novel member of the transforming growth factor-β gene family. *EMBO J.*, **6**, 3673–3677.

DERYNCK, R., JARRETT, J.A., CHEN, E.Y., EATON, D.H., BELL, J.R., ASSOIAN, R.K., ROBERTS, A.B., SPORN, M.B. & GOEDDEL, D.V. (1985). Human transforming growth factor-β complementary DNA sequence and expression in normal and transformed cells. *Nature*, **316**, 701–705.

DERYNCK, R., LINDQUIST, P.B., LEE, A., WEN, D., TAMM, J., GRAYCAR, J.Æ., RHEE, L., MASON, A.J., MILLER, D.A., COFFEY, R.J., MOSES, H.L. & CHEN, E.Y. (1988). A new type of transforming growth factor-β, TGF-β3. *EMBO J.*, **7**, 3737–3743.

EBNER, R., CHEN, R.-H., SHUM, L., LAWLER, S., ZIONCHECK, T.F., LEE, A., LOPEZ, A.R. & DERYNCK, R. (1993). Cloning of a type I TGF-β receptor and its effect on TGF-β binding to the type II receptor. *Science*, **260**, 1344–1348.

ENGELHOLM, S.A., SPANG-THOMSEN, M., VINDELØV, L.L. BRÜNNER, N., NIELSEN, M.H., HIRSCH, F. & HANSEN, H.H. (1986). Comparison of characteristics of human small cell carcinoma of the lung in patients, in vitro and transplanted into nude mice. *Acta Pathol. Microbiol. Scand., A, Pathol.*, **94**, 325–336.

FANGER, B.O., WAKEFIELD, L.M. & SPORN, M.B. (1986). Structure and properties of the cellular receptor for transforming growth factor type (beta). *Biochemistry*, **25**, 3083–3091.

GEISER, A.G., BURMESTER, J.K., WEBBINK, R., ROBERTS, A.B. & SPORN, M.B. (1992). Inhibition of growth by transforming growth factor-β following fusion of two nonresponsive human carcinoma cell lines. *J. Biol. Chem.*, **267**, 2588–2593.

INAGAKI, M., MOUSTAKAS, A., LIN, H.Y., LODISH, H.F. & CARR, B.I. (1993). Growth inhibition by transforming growth factor β (TGF-β) type 1 is restored in TGF-β-resistant hepatoma cells after expression of TGF-β receptor type II cDNA. *Proc. Natl Acad. Sci. USA*, **90**, 5359–5363.

KNABBE, C., LIPPMAN, M.E., WAKEFIELD, L.M., FLANDERS, K.C., KASID, A., DERYNCK, R. & DICKSON, R.B. (1987). Evidence that transforming growth factor-β is a hormonally regulated negative growth factor in human breast cancer cells. *Cell*, **48**, 417–428.

KOYASU, S., KADOWAKI, T., NISHIDA, E., TOBE, K., ABE, E., KASUGA, M., SAKAI, H. & YAHARA, I. (1988). Alteration of growth, cell morphology, and cytoskeletal structures of KB cells induced by epidermal growth factor and transforming growth factor beta. *Exp. Cell Res.*, **176**, 107–116.

LABARCA, C. & PAIGEN, K. (1980). A simple, rapid, and sensitive DNA assay procedure. *Anal. Biochem.*, **102**, 344–352.

LAGADEC, P.F., SARAYA, K.A. & BALKWILL, F.R. (1991). Human small-cell lung-cancer cells are cytokine-resistant but NK/LAK-sensitive. *Int. J. Cancer*, **48**, 311–317.

LAIHO, M., WEIS, F.M. & MASSAGUÉ, J. (1990*a*). Concomitant loss of transforming growth factor-β receptor types I and II in cell mutants resistant to TGF-β. *J. Biol. Chem.*, **265**, 18518–18524.

LAIHO, M., DECAPRIO, J.A., LUDLOW, J.W., LIVINGSTON, D.M. & MASSAGUÉ, J. (1990*b*). Growth inhibition by TGF-β linked to suppression of retinoblastoma protein phosphorylation. *Cell*, **62**, 175–185.

LAIHO, M., WEIS, F.M., BOYD, F.T., IGNOTZ, R.A. & MASSAGUÉ, J. (1991). Responsiveness of transforming growth factor-β restored by complementation between cells defective in TGF-β receptors I and II. *J. Biol. Chem.*, **266**, 9108–9112.

LIN, H.Y., WANG, X.-F., NG-EATON, E., WEINBERG, R.A. & LODISH, H.F. (1992). Expression cloning of the TGF-β type II receptor, a functional transmembrane serine/threonine kinase. *Cell*, **68**, 775–785.

LOPEZ-CASILLAS, F., CHEIFETZ, S., DOODY, J., ANDRES, J.L., LANE, W.S. & MASSAGUÉ, J. (1991). Structure and expression of the membrane proteoglycan, a component of the TGF-β receptor system. *Cell*, **67**, 785–795.

MASSAGUÉ, J. (1990). The transforming growth factor-β family. *Annu. Rev. Cell Biol.*, **6**, 597–641.

MASSAGUÉ, J., ANDRES, J.L., ATTISANO, L., CHEIFETZ, S., LOPEZ-CASILLAS, F., OHTSUKI, M. & WRANA, J.L. (1992). TGF-beta receptors. *Mol. Reprod. Dev.*, **32**, 99–104.

MOSES, H.L. (1992). TGF-beta regulation of epithelial cell proliferation. *Mol. Reprod. Dev.*, **32**, 179–184.

MOSES, H.L., BASCOM, C.C., COFFEY, R.J., KESKI-OJA, J., LYONS, R.M. & SIPES, N.J. (1988). Transforming growth factors in normal and neoplastic cell growth. *Prog. Cancer Res. Ther.*, **35**, 197–202.

ONG, G., SIKORA, K. & GULLICK, W.J. (1991). Inactivation of the retinoblastoma gene does not lead to loss of TGF-β receptors or response to TGF-β in breast cancer cell lines. *Oncogene*, **6**, 761–763.

PETTENGILL, O.S., SORENSON, G.D., WURSTER-HILL, D., CURPHEY, T.J., NOLL, W.W., CATE, C.C. & MAURER, L.H. (1980). Isolation and growth characteristics of continuous cell lines from small-cell carcinoma of the lung. *Cancer*, **45**, 906–918.

PIETENPOL, J.A., STEIN, R.W., MORAN, E., YACIUK, P., SCHLEGEL, R., LYONS, R.M., PITTELKOW, M.R., MÜNGER, K., HOWLEY, P.M. & MOSES, H.L. (1990). TGF-β1 inhibition of c-myc transcription and growth in keratinocytes is abrogated by viral transforming proteins with pRB binding domains. *Cell*, **61**, 777–785.

PIETENPOL, J.A., MÜNGER, K., HOWLEY, P.M., STEIN, R.W. & MOSES, H.L. (1991). Factor-binding element in the human c-myc promoter involved in transcriptional regulation by transforming growth factor β1 and by retinoblastoma gene product. *Proc. Natl Acad. Sci. USA*, **88**, 10227–10231.

ROBERTS, A.B. & SPORN, M.B. (1990). The transforming growth factor-βs. In *Handbook of Experimental Pharmacology. Peptide Growth Factors and their Receptors*, Sporn, M.B. & Roberts, A.B. (eds), pp. 419–472. Springer: Heidelberg.

RYGAARD, K., SORENSON, G.D., PETTENGILL, O.S., CATE, C.C. & SPANG-THOMSEN, M. (1990). Abnormalities in structure and expression of the retinoblastoma gene in small cell lung cancer cell lines and xenografts in nude mice. *Cancer Res.*, **50**, 5312–5317.

SMITH, P.K., KROHN, R.L. & HERMANSON, G.T. (1985). Measurement of protein using bicinichoninic acid. *Anal. Biochem.*, **150**, 76-85.

SORENSON, G.D., PETTENGILL, O.S., CATE, C.C. & DELPRETE, S.A. (1984). Biomarkers in small cell carcinoma of the lung. In *Lung Cancer*, Aisner, J. (ed.), pp. 203–240. Churchill Livingstone: New York.

SZEKELY, L., UZVOLGYI, E., JIANG, W.-Q., DURKO, M., WIMAN, K.G., KLEIN, G. & SUMEGI, J. (1991). Subcellular localization of the retinoblastoma protein. *Cell Growth Different.*, **2**, 287–295.

TEMPLETON, D.J., PARK, S.H., LANIER, L. & WEINBERG, R.A. (1991). Nonfunctional mutants of the retinoblastoma protein are characterized by defects in phosphorylation, viral oncoprotein association, and nuclear tethering. *Proc. Natl Acad. Sci. USA*, **88**, 3033–3037.

TEN DIJKE, P., HANSEN, P., IWATA, K.K., PIELER, C. & FOULKES, J.G. (1988). Identification of another member of the transforming growth factor type β gene family. *Proc. Natl Acad. Sci. USA*, **85**, 4715–4719.

TUCKER, R.F., SHIPLEY, G.D., MOSES, H.L. & HOLLEY, R.W. (1984). Growth inhibitor from BSC-1 cells closely related to type β transforming growth factor. *Science*, **226**, 705-707.

WANG, X.-F., LIN, H.Y., NG-EATON, E., DOWNWARD, J., LODISH, H.F. & WEINBERG, R.A. (1991). Expression cloning and characterization of the TGF-β type III receptor. *Cell*, **67**, 797-805.

WILDING, G., KNABBE, C., ZUGMAIER, G., FLANDERS, K. & GELMANN, E.P. (1989). Differential effects of TGFβ on human prostate cancer cells in vitro. *Mol. Cell. Endocrinol.*, **62**, 79-87.

WRANA, J.L., ATTISANO, L., CARCAMO, J., ZENTELLA, A., DOODY, J., LAIHO, M., WANG, X.-F. & MASSAGUÉ, J. (1992). TGFβ signals through a heteromeric protein kinase complex. *Cell*, **71**, 1003-1014.

WU, S., THEODORESCU, D., KERBEL, R.S., WILLSON, J.K.V., MULDER, K.M., HUMPHREY, L.E. & BRATTAIN, M.G. (1992). TGF-β1 is an autocrine-negative growth regulator of human colon carcinoma FET cells in vivo as revealed by transfection of an expression vector. *J. Cell Biol.*, **116**, 187-196.

YANAGIHARA, K. & TSUMURAYA, M. (1992). Transforming growth factor β1 induces apoptotic cell death in cultured human gastric carcinoma cells. *Cancer Res.*, **52**, 4042-4045.